# Effects of Self-Myofascial Release Using a Foam Roller on the Stiffness of the Gastrocnemius-Achilles Tendon Complex and Ankle Dorsiflexion Range of Motion

**DOI:** 10.3389/fphys.2021.718827

**Published:** 2021-09-17

**Authors:** Tian-Tian Chang, Zhe Li, Yuan-Chun Zhu, Xue-Qiang Wang, Zhi-Jie Zhang

**Affiliations:** ^1^Department of Sport Rehabilitation, Shanghai University of Sport, Shanghai, China; ^2^The First Clinical Medical School, Shaanxi University of Chinese Medicine, Xi’an, China; ^3^Department of Rehabilitation Medicine, Shanghai Shangti Orthopaedic Hospital, Shanghai, China; ^4^Rehabilitation Therapy Center, Luoyang Orthopedic Hospital of Henan Province, Luoyang, China

**Keywords:** stiffness, gastrocnemius, self-myofascial release, range of motion, ankle, foam roller

## Abstract

Increased muscle stiffness can contribute to reduced range of motion (ROM) and impaired function. Reduced ankle dorsiflexion ROM has been associated with increased injury risk in the ankle. Self-myofascial release (SMR) has been widely used in clinical and sports settings, but the effects of SMR on gastrocnemius and Achilles tendon (AT) stiffness are unclear. Therefore, we investigated the effects of self-myofascial release using a foam roller (FR) on the stiffness of the gastrocnemius–AT complex and ankle dorsiflexion ROM. Fifty healthy, untrained, and non-sedentary participants (age=22.5±2.6years) were randomly divided into an intervention group (FR group) and a control group. The subjects in the intervention group received a single foam roller intervention (three sets of 1min), while the subjects in the control group performed a 5-min sedentary rest. Stiffness of the gastrocnemius–AT complex was evaluated using MyotonPRO and the ankle dorsiflexion ROM was assessed using the weight-bearing lunge test. For the foam roller and control groups, the between-group analysis revealed a statistically significant difference in gastrocnemius stiffness and ankle dorsiflexion ROM after intervention (*p*<0.05). Within-group analysis revealed a significant increase in ROM and a significant decrease in medial and lateral gastrocnemius (LG) stiffness for the foam roller group after the intervention (*p*<0.05). In addition, further analysis of the preintervention data revealed a significant negative correlation between ankle dorsiflexion ROM and AT stiffness (*r*=−0.378 and *p*=0.007). These results suggest that self-myofascial release using a foam roller on the calf is an effective method for decreasing the stiffness of the gastrocnemius and increasing ankle dorsiflexion ROM.

## Introduction

Stiffness is one of the mechanical properties of muscle that can affect exercise capacity and has been correlated with muscle strain injury risk, especially in high-intensity activities (Witvrouw et al., 2004; Watsford et al., 2010). The muscle and tendon may also play an important role in passive range of motion (ROM; Hirata et al., 2020). It has been shown that increased muscle stiffness (i.e., resistance to stretching) can contribute to reduced ROM and impaired function (Geertsen et al., 2015). In addition to these contractile structures, non-muscular structures (e.g., nerves and fasciae) can limit passive ankle dorsiflexion ROM (Andrade et al., 2016; Nordez et al., 2017). Reduced ankle dorsiflexion ROM has been associated with increased injury risk in both acute and overuse injuries to the ankle joint and surrounding tissues (e.g., sprains, Achilles tendinopathy, patellar tendinopathy, and general lower extremity pain; Malliaras et al., 2006; Whitting et al., 2013; Rabin et al., 2014; Knapik et al., 2019). Thus, optimal methods of decreasing the muscle stiffness and increasing ankle dorsiflexion ROM for the prevention of injury need to be identified.

In recent years, self-myofascial release (SMR) has been widely used in clinical and sports settings. SMR is a self-treatment method involving the application of compressive forces to soft tissue. It claims to mimic the effects of manual therapy techniques and aims to address soft tissue dysfunction (Krause et al., 2017; Wilke et al., 2020). The foam roller (FR), a device commonly used for self-myofascial release, is also a popular device in sports and physical therapy (De Benito et al., 2019). An advantage of the FR is that it usually does not impair subsequent muscle strength (Madoni et al., 2018) and jump height (Wiewelhove et al., 2019). Moreover, foam roller use can also improve sprint performance (DʼAmico and Gillis, 2019), reduce muscle pain (Macdonald et al., 2014), and improve neuromuscular efficiency (Bradbury-Squires et al., 2015). To illustrate the effects of SMR, some studies have primarily focused on how foam roller use affects joint ROM, soreness, and lower extremity mechanical properties (Laffaye et al., 2019). Although, some studies have found that use of a foam roller can increase joint ROM, little is known about the mechanism by which this increase in ROM occurs (Wiewelhove et al., 2019). In addition, some studies have focused on the effect of foam roller on stiffness of soft tissue in the posterior or anterior thigh (Morales-Artacho et al., 2017; Krause et al., 2019; Wilke et al., 2020). And limited studies have explored the acute effect of foam roller on the stiffness of the gastrocnemius (Kiyono et al., 2020; Nakamura et al., 2021). Nevertheless, there is almost no study exploring the effect of foam roller on the Achilles tendon (AT) stiffness. Therefore, it is necessary to understand variations in gastrocnemius and AT stiffness after foam roller use to improve our understanding of the effect of self-myofascial release and to provide a reference for the application of and further clinical research on self-myofascial release. Understanding the factors affecting ROM is also one of the important topics in clinical and research fields (Nordez et al., 2017). To the best of our knowledge, limited studies have reported the correlation between muscle stiffness and joint ROM (Miyamoto et al., 2018; Hirata et al., 2020), and no study has explored the correlation between tendon stiffness and joint ROM. The exact correlation between ankle dorsiflexion ROM and gastrocnemius or Achilles tendon stiffness, therefore, awaits further investigation.

In recent years, MyotonPRO, a hand-held device, has been used to quantify the stiffness of soft tissues. Although, soft tissue stiffness measured by MyotonPRO is similar to Young’s modulus evaluated by shear wave elastography, neither of these is equivalent to the true elasticity of the modulus obtained from *in vitro* biomechanical testing; however, they can reflect the relative stiffness of soft tissues (Kelly et al., 2018). The MyotonPRO has been proved to have a good intra- and interrater reliability in assessing gastrocnemius and AT stiffness (Feng et al., 2018; Liu et al., 2018). More importantly, we found a significant correlation between the stiffness and shear modulus of the gastrocnemius and AT as quantified by a MyotonPRO and shear wave elastography, respectively (Feng et al., 2018). Therefore, we used a MyotonPRO to quantify changes in gastrocnemius and AT stiffness.

The purposes of this study were to investigate (1) the acute effect of self-myofascial release using the foam roller on the stiffness of the gastrocnemius and AT; (2) the acute effect of a foam roller intervention on passive ankle dorsiflexion range of motion; and (3) the associations of passive ankle dorsiflexion ROM with gastrocnemius and AT stiffness.

## Materials and Methods

### Participants

Fifty healthy, untrained, and non-sedentary participants (25 males and 25 females) were recruited for the present study. The inclusion criteria were as follows: (1) age range: 18–35years; (2) Body mass index (BMI) range: 16–28kg/m^2^; (3) no known cardiovascular, pulmonary, or neurological diseases; and (4) no skin lesions in the lower limbs. Participants were excluded if they met the following criteria: (1) acute or chronic musculoskeletal system diseases or neuromuscular diseases; (2) any gastrocnemius or Achilles tendon dysfunction; (3) a history of musculoskeletal injuries to the ankle joint (e.g., ankle sprain) within the previous 6months; (4) a history of surgery in the lower extremities; or (5) inability to complete the whole experiment. Fifty participants were randomly divided into an intervention group (foam roller group, 13 males and 12 females) and a control group (12 males and 13 females).

### Procedures

About 1week before the testing sessions, participants attended a standardized familiarization session including the testing procedure and the foam roller intervention to minimize learning effects. All tests were performed in the dominant limb in a room with a temperature of 25°C. The demographic characteristics (age, sex, height, and weight) and the dominant limb were recorded when participants arrived at the testing area. The dominant limb was defined by the side the participant used when asked to kick a ball (Lenskjold et al., 2015; Zhang et al., 2015). To further minimize error, we measured the stiffness of the soft tissue and then the ankle dorsiflexion ROM prior to the intervention. The average of three successive stiffness and ROM measurements was calculated in this study. The subjects in the intervention group received a single foam roller intervention, while the subjects in the control group performed a 5-min sedentary rest.

### Foam Roller Exercises

**Figure 1:**
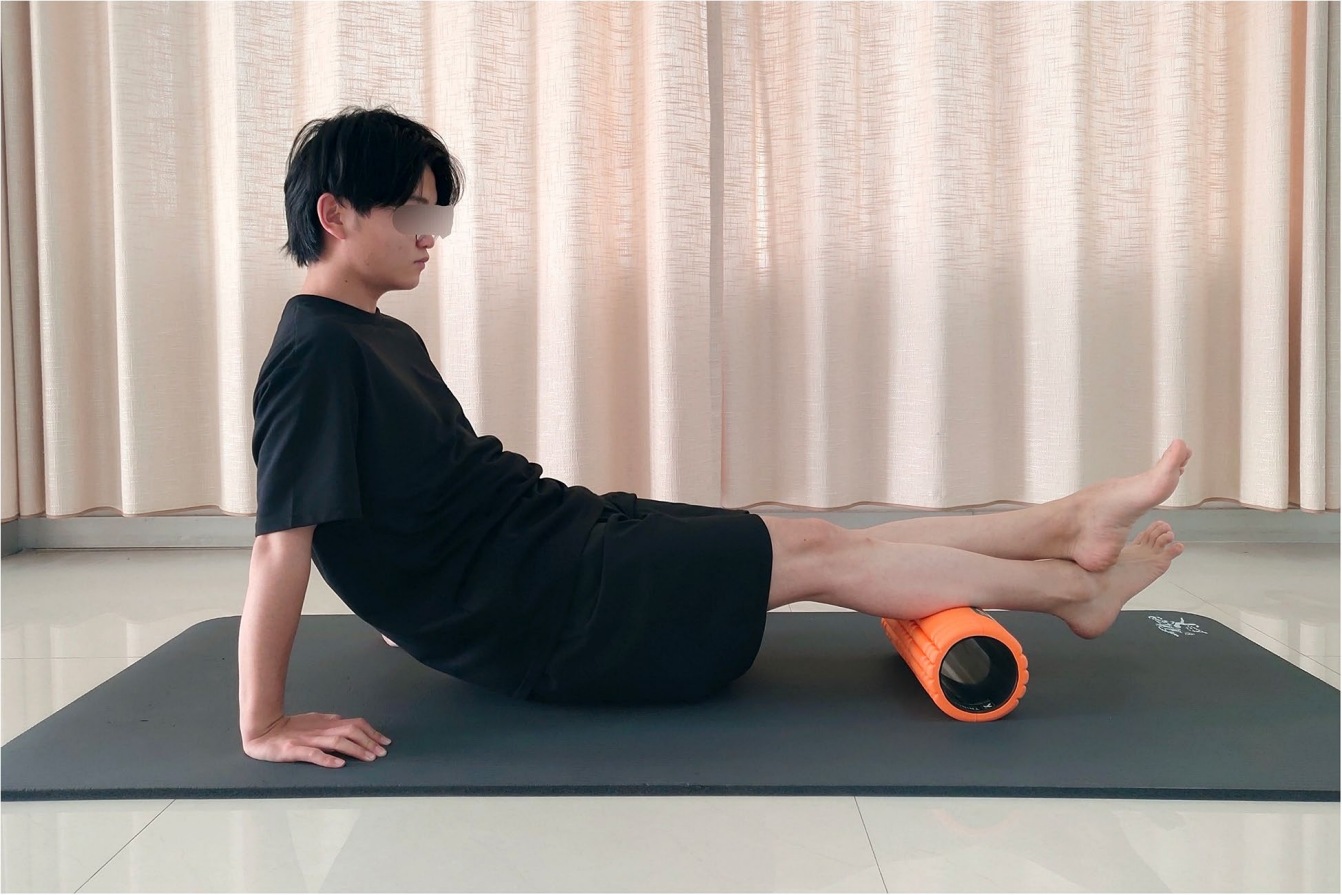
Foam roller (FR) exercises.

In this study, FR exercises were conducted on the calf of the subject’s dominant limb with a GRID Foam Roller (The GRID foam roller; height 33cm; diameter 14cm; Muller Japan Co., Yokohama, Japan). According to a previous study, the GRID is composed of a hollow inner core enclosed in a 15mm thick layer of ethylene-vinyl acetate foam, with a total Young’s modulus of 103kPa (Yoshimura et al., 2019). For the FR exercises, the participants were in an adapted seated position with their dominant calf resting on the FR and the nondominant leg crossed over the dominant leg, using their hands to elevate the trunk and keep the gluteals off the ground (Figure 1). The participants were instructed to use their arms to propel their body forward and back to perform the FR intervention between the popliteal fossa and Achilles tendon. Based on previous studies (Krause et al., 2019; Kiyono et al., 2020), participants were instructed to subjectively control the pressure on the calf to a 7/10, as measured by the numerical rating scale, with 0/10 indicating no discomfort and 10/10 representing maximum discomfort. All the participants in the intervention group completed three sets of 1min FR exercises with a 30s rest between sets (Yoshimura et al., 2019). Each set consisted of 20 movement cycles, with one proximal rolling plus one subsequent distal rolling movement counted as one movement cycle. A metronome was used to standardize the rolling rate in this study.

### Ankle Dorsiflexion ROM

The ankle dorsiflexion ROM of the dominant limb was measured using the weight-bearing lunge test (Kelly and Beardsley, 2016; Somers et al., 2020). The weight-bearing lunge test has been shown to have a high reliability (ICC>0.97; Bennell et al., 1998). And they indicated that the every 1cm away from the wall is equivalent to approximately 3.6° of ankle dorsiflexion (Bennell et al., 1998). We measured the ankle dorsiflexion ROM of the dominant limb for all participants in this study. Subjects placed their dominant foot on a ruler fixed to the ground and stood with their foot approximately 10cm away from and perpendicular to the wall. The participants were then instructed to lunge forward, flexing their knee until their knee touched the wall. If the knee touched the wall and the heel remained firmly on the ground, it was considered a successful maneuver (Škarabot et al., 2015). The subjects were then instructed to move their foot back and attempt to touch their knee to the wall again if their knee had successfully touched the wall in the previous attempt. Conversely, if their knee failed to touch the wall in the previous test, subjects were instructed to move their foot forward and attempt again. This process was repeated until their knee was just touching the wall with the heel on the ground, indicating the limit of their ankle ROM. We measured the distance between their great toe and the wall at the limit of their ROM. After the FR intervention, we immediately measured soft tissue stiffness and then ankle dorsiflexion ROM.

### Stiffness Measurements

A MyotonPRO (Myoton AS, Tallinn, Estonia) was used to quantify the stiffness of the medial gastrocnemius (MG), lateral gastrocnemius (LG), and AT in the present study. In our previous studies, we found a good intra- and inter-rater reliability (ICC>0.84) for assessing the MG, LG, and AT stiffness using the MyotonPRO (Liu et al., 2018; Chang et al., 2020). The basic principles of the MyotonPRO are described as follows: mechanical impulses can cause oscillations of soft tissues following the probe of the MyotonPRO precompressing the assessed soft tissues; the accelerometer of the MyotonPRO measures the mechanical oscillations of assessed soft tissues; and five parameters of the assessed tissue are obtained using information about the subsequent oscillations. Stiffness is one of these mechanical property parameters. The device measures the stiffness value in Newtons/meter (N/m), with a larger value indicating stiffer tissue.

The MG measurement site was located at 30% of the length between the popliteal fossa and lateral malleolus, where cross-sectional areas of the gastrocnemius are almost maximum (Hirata et al., 2017). LG stiffness was measured at one-third of the length between the small head of the fibula and the heel (Masood et al., 2014). The measurement site of AT stiffness was 4cm proximal to the tendon insertion (calcaneal tuberosity) because Achilles tendinopathy is commonly seen in this area (Stenroth et al., 2012). Similar to previous studies, stiffness measurements were performed when participants were in a prone position with the knee joint fully extended and the hip in the neutral position (Chino and Takahashi, 2018; Liu et al., 2018). The stiffness of the MG, LG, and AT was evaluated with a MyotonPRO with the ankle joint in a relaxed position (DeWall et al., 2014; Morgan et al., 2018). The probe of the MyotonPRO was placed perpendicular to the surface of the soft tissue for the stiffness measurement.

### Statistical Analysis

All statistical analyses were performed using SPSS software (SPSS version 22.0, IBM, United States). Descriptive data and all stiffness data are presented as the mean±SD. The Shapiro-Wilk test was used to assess the normal distribution of all data. Homogeneity of variances was evaluated using Levene’s test. BMI was calculated by the following formula: BMI=weight (kg)/height (m^2^). A paired *t*-test was used to examine the differences pre- to postintervention for ROM, gastrocnemius stiffness, and AT stiffness in the control group and intervention group. The differences in gastrocnemius stiffness, AT stiffness, and ankle dorsiflexion ROM between the foam roller and control groups were compared using an independent sample *t*-test. Pearson correlation analysis (r) was used to analyze the correlation among gastrocnemius stiffness, Achilles tendon stiffness, and ROM. In addition, the effect size was also calculated using Cohen’s d (Cohen, 1998). Cohen’s d values less than 0.2, 0.2–0.5, and greater than 0.8 correspond to small, medium, and large effects, respectively. The significance level was set at *p*<0.05 for all tests.

## Results

### Demographic Data

Demographic information, including age, height, and BMI, for all subjects is shown in Table 1.

**Table 1:** The characteristics of the subjects.

	Foam roller group	Control group	*p*
Age (years)	22.5±2.4	22.4±2.9	0.958
Height (m)	1.69±0.08	1.68±0.08	0.903
Weight (kg)	63.8±13.0	64.5±13.4	0.848
BMI (kg/m^2^)	22.3±3.3	22.6±3.0	0.766

### Variations in LG, MG, and AT Stiffness in Both the Foam Roller and Control Groups

**Figure 2:**
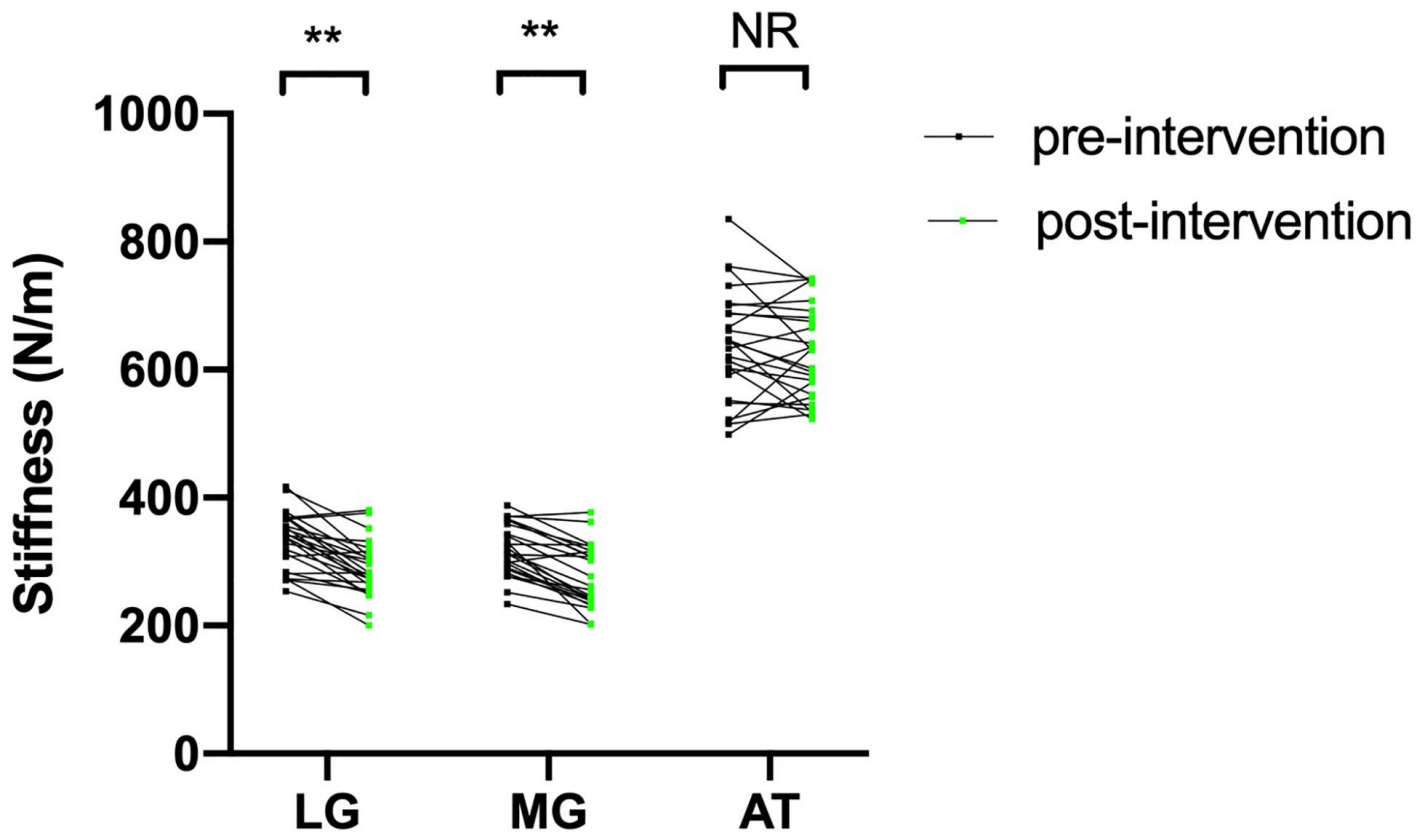
The change in stiffness of the LG, MG, and AT in the FR group before and after foam roller intervention. FR, foam roller; MG, medial gastrocnemius; LG, lateral gastrocnemius; and AT, Achilles tendon; ^**^p<0.01. NS, nonsignificant at *p*>0.05.

Table 2 reveals the LG, MG, and AT stiffness in both the foam roller and control groups before and after the intervention. There was no significant difference in LG, MG, or AT stiffness between the foam roller and control groups preintervention (*p*>0.05). After the intervention, the LG and MG stiffness in the foam roller group was significantly lower than that in the control group (*p*<0.05), while there was no statistical difference in AT stiffness between the foam roller and control group (*p*>0.05). As shown in Figure 2 and Table 2, LG and MG stiffness in the FR group decreased significantly after foam roller intervention. However, there was no significant change in AT stiffness in the FR group after the intervention (*p*>0.05).

**Table 2:** Changes in LG, MG, and AT stiffness and ankle dorsiflexion ROM before and after the intervention.

		Foam roller group M±SD	Control group M±SD	*p*	Cohen’s d
LG	Pre(N/m)	333.8±43.6	334.4±46.5	0.963	--
	Post(N/m)	288.4±43.6	330.4±47.9	0.002^**^	0.917
	% Change	−13.3±10.1	−1.0±7.7	0.001^**^	1.370
MG	Pre(N/m)	319.2±40.4	322.8±43.5	0.760	--
	Post(N/m)	276.8±48.5	320.8±52.0	0.003^**^	0.875
	% Change	−13.3±9.4	−0.8±7.1	0.001^**^	1.500
AT	Pre(N/m)	637.8±85.5	645.5±74.6	0.736	--
	Post(N/m)	626.3±73.1	652.0±78.4	0.238	--
	% Change	−1.2±9.4	1.0±3.7	0.284	--
ROM	Pre(cm)	14.4±0.8	14.5±1.1	0.720	--
	Post(cm)	16.1±1.0	14.5±1.1	0.001^**^	1.509
	% Change	11.4±4.8	−0.3±1.4	0.001^**^	3.309

Pre, before the foam roller intervention; Post, after the foam roller intervention; CI, confidence interval; ROM, range of motion; MG, medial gastrocnemius; LG, lateral gastrocnemius; and AT, Achilles tendon;

** *p*<0.01.

### Variations in Ankle Dorsiflexion ROM in Both the Foam Roller and Control Groups

**Figure 3:**
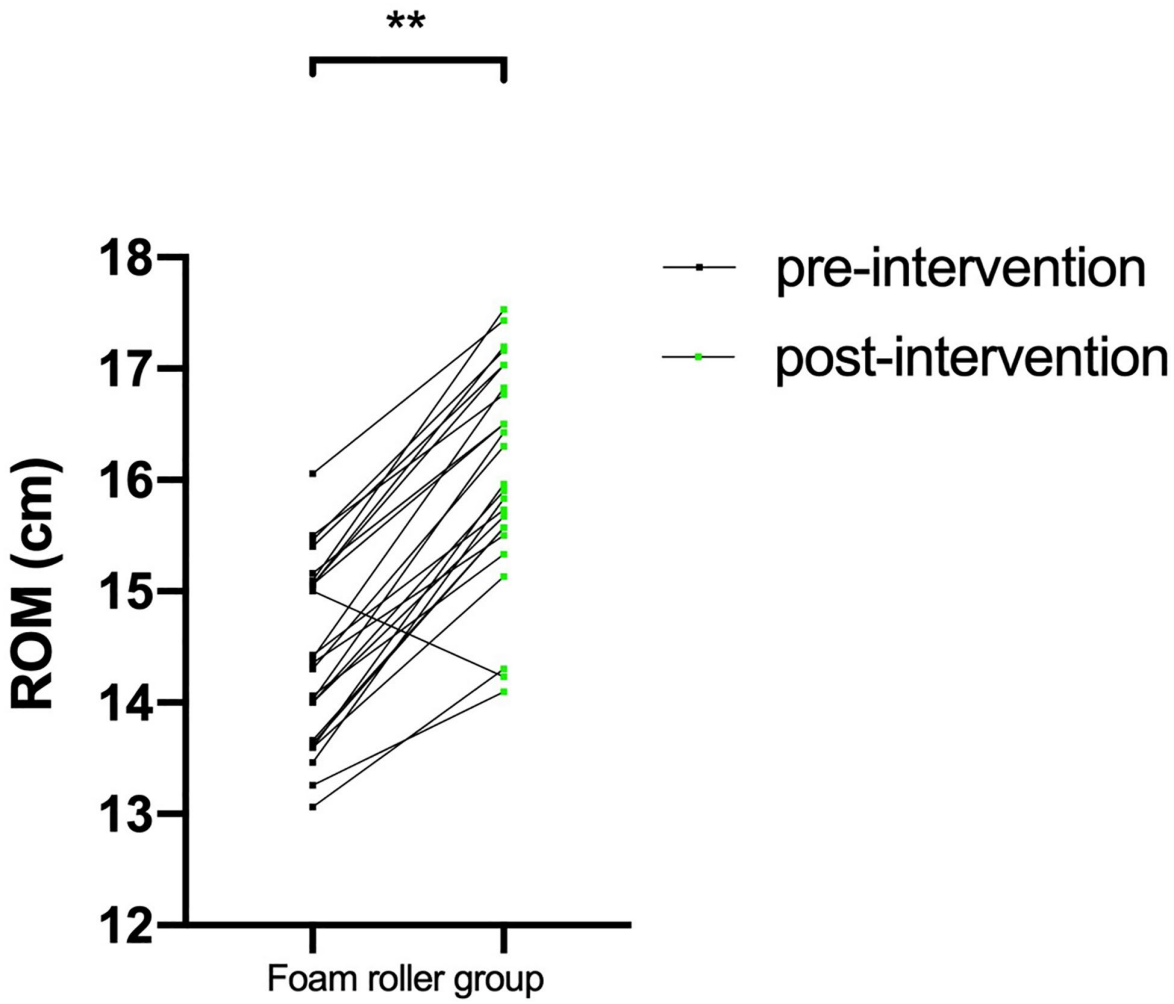
The change in ankle dorsiflexion ROM in the FR group before and after foam roller intervention. FR, foam roller; ROM, range of motion; ^**^p<0.01.

The ankle dorsiflexion ROM of the foam roller and control groups is presented in Table 2. No significant between-group differences were observed in ankle dorsiflexion ROM at preintervention. The ankle dorsiflexion ROM in the foam roller group was greater than that in the control group following intervention (*p*<0.05). There was a significant increase in ankle dorsiflexion ROM in the FR group after the intervention (*p*<0.05; Figure 3).

### The Relationship Between Ankle ROM and LG, MG, and AT Stiffness

**Figure 4:**
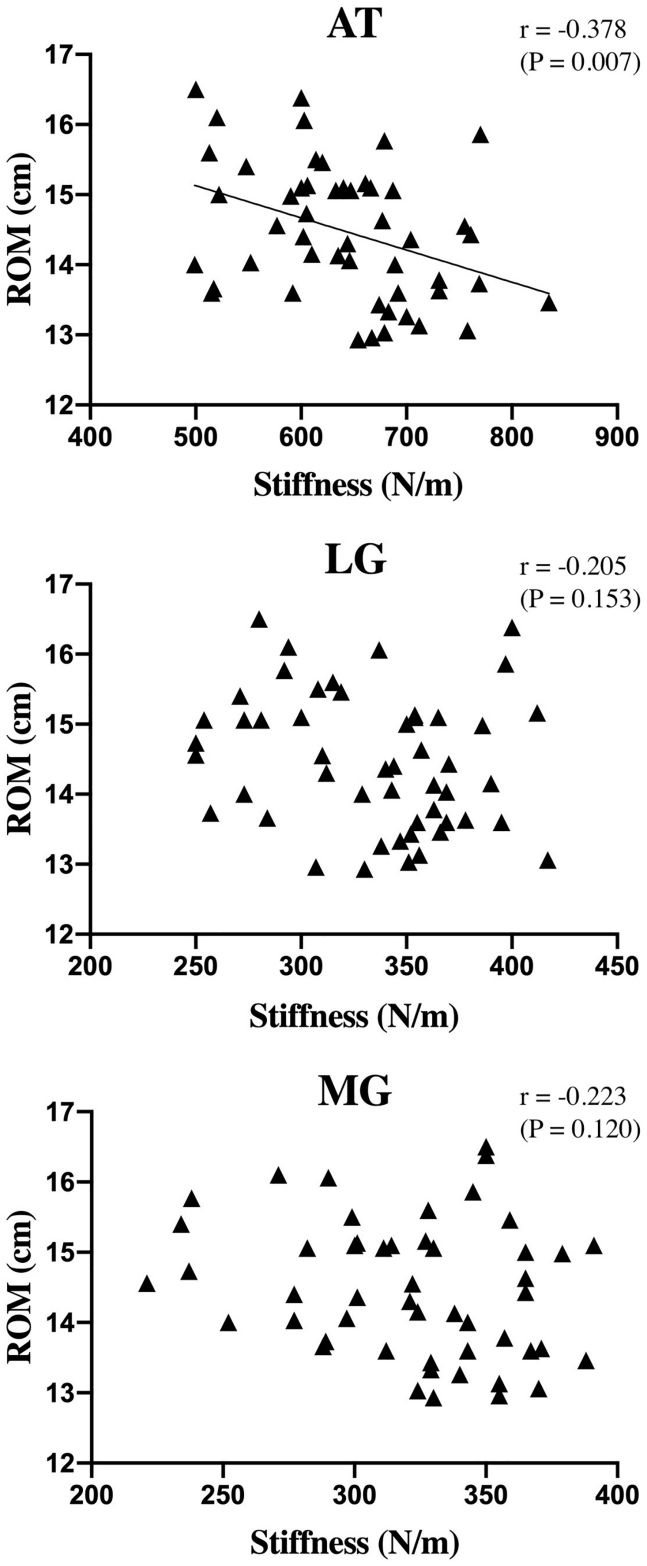
Relationship of ankle dorsiflexion ROM and MG, LG, and AT stiffness. r, correlation coefficient; ROM, range of motion; MG, medial gastrocnemius; LG, lateral gastrocnemius; and AT, Achilles tendon.

Figure 4 shows the relationships between ankle dorsiflexion ROM and LG, MG, and AT stiffness prior to the intervention. Further analysis of the preintervention data revealed a negative correlation between AT stiffness and ankle dorsiflexion ROM (*r*=−0.378 and *p*=0.007). However, for muscle, no significant correlation was apparent between MG stiffness and ankle dorsiflexion ROM (*p*>0.05). Similarly, there was no significant correlation between LG stiffness and ankle dorsiflexion ROM (*p*>0.05). After the FR intervention, no significant correlation was apparent between the ankle dorsiflexion ROM and LG, MG, AT in the FR group (the correlation coefficients ranged from −0.232 to −0.338, all *p*>0.05).

## Discussion

The present study investigated gastrocnemius stiffness, AT stiffness, and ankle dorsiflexion ROM before and after an intervention in foam roller and control groups. Accordingly, our results showed a significant decrease in gastrocnemius stiffness and a significant increase in ankle dorsiflexion ROM only in the foam roller group, with no significant change in AT stiffness. In addition, there was no correlation between ankle dorsiflexion ROM and LG and MG stiffness, but a significant negative correlation was observed between ankle dorsiflexion ROM and AT stiffness.

### LG, MG, and AT Stiffness in Both the Foam Roller and Control Groups

The results of this study showed that gastrocnemius stiffness significantly decreases after the foam roller intervention, whereas there was no significant change in the Achilles tendon stiffness. A recent study investigated stiffness variation in the hamstring muscles after performing a foam roller protocol using shear wave elastography, and the authors found a significant decrease in average hamstring muscle stiffness 5min after FR (Morales-Artacho et al., 2017). Reiner et al. (2021) compared the effect of foam rolling and vibration foam rolling applied for 3min on the stiffness of the quadriceps muscle. And they demonstrated an immediate decrease in rectus femoris stiffness in both the vibration group and the non-vibration group. Wilke et al. (2019) compared the effects of high-velocity and slow-velocity foam roller use to the anterior thigh; they found that the anterior thigh tissue stiffness decreased immediately regardless of velocity, and there was no difference in stiffness variation between the two groups. In contradiction to our findings, Kiyono et al. (2020) and Nakamura et al. (2021) found no significant change in MG stiffness before and after FR intervention. Such conflicting results may due to the different measurement methods. In the studies of Kiyono et al. (2020) and Nakamura et al. (2021), the MG stiffness was measured at 10° dorsiflexion of the ankle. In the study of Nakamura et al. (2021), the participants in the three groups received the FR intervention for 30, 90, and 300s, respectively. The participants underwent a 5-week foam rolling intervention (three times per week, 90s each time) with at least 24–48h of rest between each intervention in the study of Kiyono et al. (2020). But the subjects performed a single foam roller intervention (three sets of 1min) in our study. Beyond that, Morales-Artacho et al. (2017) suggested that the reduction in stiffness after FR intervention must be considered a short-term effect, as its value returns to baseline within 15min. In this study, we immediately measured the soft tissue stiffness after the FR intervention. But in the study by Kiyono et al. (2020), they did not state the measurement time of MG stiffness. As stated before, the stiffness measurement time and the foam rolling program (i.e., intervention intensity) may account for the discrepancies in the findings. There was no significant change in AT stiffness in the foam roller group after the intervention in this study. Similarly, Konrad and Tilp (2020) found that although the ankle dorsiflexion ROM increased after a 3-min static stretching exercise, there is no significant change in AT stiffness.

The decrease in muscle stiffness is one of the proposed mechanisms for acute morphological responses after SMR (Ryan et al., 2008). Foam rolling induces pressure and friction on the treated muscle, skin, and fascia, and compression of muscle and surrounding fascial tissues may stimulate the activity of contractile cells, affecting tissue hydration or the mechanical properties of muscle fibers, thereby altering the stiffness of the tissue (Chaudhuri et al., 2007). Stable cross-bridges, formed between actin and myosin, are also thought to be one of the factors affecting resting muscle stiffness (Proske and Morgan, 1999; Eriksson Crommert et al., 2015). Morales-Artacho et al. (2017) suggested that active or passive mobilization (including foam roller interventions) of the lower limb may cause cross-bridge release and thus reduce muscle stiffness.

### Ankle Dorsiflexion ROM in Both the Foam Roller and Control Groups

In the present study, our findings revealed an increase in ankle dorsiflexion ROM with 11.44% in the foam roller group from pre- to postintervention. Škarabot et al. (2015) reported that the effect of static stretching on ankle dorsiflexion ROM in adolescent athletes (five females, six males). And they found the ankle dorsiflexion ROM increased by 6.2% (from 14.5 to 15.4cm) after a single static stretching (three sets of 30s). De Benito et al. (2019) demonstrated that use of a foam roller (two sets of 60s applications with 30s rest) assists in the improvement of ankle dorsiflexion ROM. Interestingly, foam roller intervention on one lower limb can increase ankle dorsiflexion ROM in the stimulated lower limb and additionally may produce a crossover effect on the contralateral limb (Kelly and Beardsley, 2016). In our study, the differences in mean values of ankle dorsiflexion ROM between the preintervention and postintervention in foam roller group exceeded the minimal detectable change of 1.1cm (Konor et al., 2012), suggesting that the difference in measurements is a real difference. Overall, FR interventions are considered the clinical intervention of choice for increasing ROM in large muscle groups (Wilke et al., 2019; Reiner et al., 2021).

There are many theories that attempt to explain the increased ROM after foam roller interventions. Some scholars have suggested that SMR can positively affect fascial sliding properties by eliminating fascial restriction or loosening crosslinks (Wiewelhove et al., 2019). Kiyono et al. (2020) and Nakamura et al. (2021) explained that the increase in ankle dorsiflexion ROM after SMR using a foam roller may be due to an increase in pain threshold (i.e., stretch tolerance). Another hypothesis for the increased ROM is that tissue stiffness changes after foam roller intervention (Ryan et al., 2008). Wilke et al. (2020) indicated that the reduction in tissue stiffness associated with increased ROM also applies to an acute bout of foam rolling. In addition, neurological modulation (autonomic nervous system response) may explain the increased ipsilateral ROM and the crossover effect after foam roller intervention (Bradbury-Squires et al., 2015; Yoshimura et al., 2019).

### The Relationship Between ROM and LG, MG, and AT Stiffness

In this study, we found no significant correlation between ankle dorsiflexion ROM and LG or MG stiffness, but ankle dorsiflexion ROM was negatively correlated with AT stiffness. Magnusson et al. (1997) compared the passive torque-joint angle relationship in inflexible and flexible participants and found that subjects with less ROM had a stiffer muscle. However, Miyamoto et al. (2018) mentioned that it is impossible to evaluate passive muscle stiffness from the results of the passive torque-angle relationship. And they found no significant correlation between MG stiffness (measured at the ankle neutral position and the muscle slack angle) or LG stiffness (measured at the muscle slack angle) evaluated by the shear wave elastography and ankle dorsiflexion ROM. However, they did not explain the possible reason for that. Miyamoto et al. (2018) and Hirata et al. (2020) demonstrated that MG and LG stiffness (evaluated at 15° and 14° dorsiflexion of the ankle joint) was negatively correlated with passive ankle dorsiflexion ROM in young men. Above evidences indicated that the associations between ankle dorsiflexion ROM and passive muscle stiffness may be different in different ankle positions.

To the best of our knowledge, this is the first study to explore the relationship between ankle dorsiflexion ROM and AT stiffness. We found a negative correlation between ankle dorsiflexion ROM and AT stiffness, but not muscle stiffness. However, the reason for this is still unclear. The possible explanation may due to different the elastic properties of muscle and tendon. Furthermore, ankle dorsiflexion ROM may be associated with various factors (Geertsen et al., 2015; Andrade et al., 2016; Nordez et al., 2017). Apart from the stiffness of the muscle and tendon, the elasticity of non-muscular tissues such as nerve and fascia has recently been proposed to influence joint flexibility (Nordez et al., 2017; Andrade et al., 2018). However, we only evaluated the muscle and tendon stiffness in this study. Further studies will be conducted to investigate these points.

### Limitations

Some limitations to this study should be recognized. First, we evaluated the stiffness variations in specific regions of the gastrocnemius and Achilles tendon, but this is not representative of changes in other regions. Second, we did not record the variation in stiffness and ankle dorsiflexion ROM with time after the foam roller intervention. In addition, we did not compare the effects of foam roller intervention with other common interventions in clinical practice, such as static stretching. In this study, we measured the stiffness of gastrocnemius and Achilles tendon in the relaxed position of the ankle. However, the resting ankle angle varies from person to person and may have some influence on these results. Finally, we performed only one foam roller intervention protocol and did not explore the effects of different FR intervention intensities on the gastrocnemius-Achilles tendon complex stiffness and ankle dorsiflexion ROM. Therefore, future studies should investigate the effects of different FR durations and frequencies on soft tissue stiffness and ankle dorsiflexion ROM or other functional outcomes.

## Conclusion

We conclude that a single foam roller intervention on the calf can increase the ankle dorsiflexion ROM and reduce the stiffness of the gastrocnemius. The present study suggests a negative correlation between ankle dorsiflexion ROM and AT stiffness in the healthy population.

## Data Availability Statement

The raw data supporting the conclusions of this article will be made available by the authors, without undue reservation.

## Ethics Statement

This study was approved by the Human Subjects Ethics Committee of Luoyang Orthopedic Hospital of Henan Province (2019-001-01). Experimental procedures conformed to the Declaration of Helsinki principles and national guidelines. The patients/participants provided their written informed consent to participate in this study.

## Author Contributions

T-TC designed the study and drafted the manuscript. Z-JZ and XQ-W helped to conceive the study. ZL and Z-JZ helped to perform statistical analysis. T-TC, ZL, and Y-CZ participated in the data collection. All authors contributed to the article and approved the submitted version.

## Conflict of Interest

The authors declare that the research was conducted in the absence of any commercial or financial relationships that could be construed as a potential conflict of interest.

## Publisher’s Note

All claims expressed in this article are solely those of the authors and do not necessarily represent those of their affiliated organizations, or those of the publisher, the editors and the reviewers. Any product that may be evaluated in this article, or claim that may be made by its manufacturer, is not guaranteed or endorsed by the publisher.
